# Artificial Intelligence and Virology - quo vadis

**DOI:** 10.6026/97320630013410

**Published:** 2017-12-31

**Authors:** Paul Shapshak, Charurut Somboonwit, John T. Sinnott

**Affiliations:** 1 Division of Infectious Diseases and International Health, Department of Internal Medicine, University of South Florida, Morsani College of Medicine, Tampa, FL 33606, USA; 2 Division of Infectious Diseases and International Health, Department of Internal Medicine, Tampa General Hospital, University of South Florida, Morsani College of Medicine, Tampa, FL 33606, USA; 3 Clinical Research Unit, Hillsborough Health Department, Tampa, Florida 33602, USA

**Keywords:** Infectious diseases, virology, R&D, Artificial Intelligence (AI), robotics, co-robotics (cobots), quantum computers (QC), cybernetics, thinking machines, repair machines, self-repair machines, bioinformation, bioinformatics, psychology machines

## Abstract

Artificial Intelligence (AI), robotics, co-robotics (cobots), quantum computers (QC), include surges of scientific endeavor to produce machines (mechanical and software) among numerous types and constructions that are accelerating progress to defeat infectious diseases. There is a plethora of additional applications and uses of these methodologies and technologies for the understanding of biomedicine through bioinformation discovery. Therefore, we briefly outline the use of such techniques in virology.

## Discussion

AI and its antecedent concepts sprang upon the scientific scene during the latter part of the 20th century. As remarked in a previous publication [1], Vannevar Bush first discussed the use of machines to augment human comprehension in 1945 [2], Alan Turing proposed machines that could simulate human intellect and thinking in 1950 [3], and the term, AI, was first used by John McCarthy in 1956 [4, 5]. By 1950, Norbert Wiener had envisioned future societies with global communication systems, intelligent computers, and interactions among computers and people [6]. John von Neumann worked heavily using computers and proposed research into intelligent and self-replicating machines [7]. Moreover, in 1955, John Nash proposed thinking machines with complex concepts to solve seemingly undecipherable problems [8]. Since the latter part of the 20th century, the requirements of computer power to support thinking computers commenced to far surpass the capabilities of classical computer design. In 1984, Richard Feynman addressed this problem and proposed the application of the laws of quantum mechanics to accelerate the speed, capacity, and calculating power of computers [9, 10]. This heralded the arrival of quantum computers.

An important application of the idea that machines can mimic and surpass human capabilities is robotics. Robotics has a nebulous history of progress and development over the last few millennia [11]. Recently, however, there has been an upsurge of interest in co-robots (cobots), a new field that brings to the fore, interactions of robots among people as well as among other robots. In this respect, studies are in progress to determine conditions for enhancement of human activities using cobots of all sorts, construction, and objectives. Hypotheses and questions examine if cobots can sense, design, scheme, exploit, and acquire knowledge in ambiguous, actual, everyday settings, circumstances, and milieus. Can they efficaciously cooperate with diverse people and with other robots? Can they do so, on the one hand, in closely-knit organizations and, on the other hand, in scattered and variegated schemes? Can scientists in the field, enable far-reaching, anodyne, vigorous, and dependable cobots that can maneuver in environs that are composite and multifaceted? These are some of the questions, goals, and hypotheses raised at the inception and implementation of such undertakings [12]. The international Federation of Robotics exemplifies the current global expansion of robotic and cobot research and development [13].

Given such questions and growth, cobots could decidedly be helpful adjuncts for the virologist, be it in the laboratory, research, development, exploring environments that contain highly infectious diseases, as well as for clinical applications. Further, the use of cobots for data assessment, discussions with their human professional associates, and scientific analysis of results and prognosis are included in this design. Besides, will professionals welcome the use of such super-intelligence and skill to improve speed, accuracy, accomplishment, and purpose? Cobots would use optimized strategically interactive AI, machine-learning, and perhaps even provide instant access to specialized 'CLOUDs' mainly created with dedicated rapidlyfunctioning databases, engineered and licensed for these purposes.

In addition, above and beyond these approaches and caveats, can cobots and professional practitioners adjust to each other within the usual norms of behavior coupled with enunciated language? Blocks, obstructions, and obstacles could occur; the learning process for both scientists and physicians with their cobots would be a necessary development in the practice and evolution of virology specifically, and in the sciences and medicine, overall. Each becomes educated about the other; AI and software professionals would continually need to improve, enhance, and optimize their work in such systems. Moreover, as the intelligence of AI increases, special robots and cobots would become psychologists, necessary to optimize further progress and evolution.

## Conclusions

AI, cobots, and QC exemplify development of technologies in the mechanical and software realms that are accelerating advances in the defeat of animal and human infectious diseases, virology, and community medicine. The vector trajectories of these technologies increasingly help animal and human health. Animals as well as humans are included in this analysis because of the close relationships and diseases that are shared among animals and humans. Throughout human evolution and history, animals have been reservoirs of diseases that impact humans. A few basic examples are viruses including Ebola, influenza, syphilis, and HIV that originate in a variety of animals resulting in human infections [14, 15]. Therefore, the scope and advancement of AI and the new technologies mentioned in this brief review require inclusion of applications to animals as well as to humans. Education of cobots as well as scientists and physicians will evolve through practice, communication, and skill. This defines in part our future, with omnipresent cobots, scientists, and clinical professionals. Products of such environments will require the exploration and assessment of legitimate, principled, as well as commercial ramifications. Their application and use will help improve communities, environment, health, and save lives.
